# Health Priorities in French-Speaking Swiss Cantons

**DOI:** 10.15171/ijhpm.2017.91

**Published:** 2017-07-31

**Authors:** Philippe Chastonay, Jean Simos, Nicolas Cantoreggi, Rudolf Zurkinden, Thomas Mattig

**Affiliations:** ^1^ University of Fribourg, Fribourg, Switzerland.; ^2^ Institute of Global Health, University of Geneva, Geneva, Switzerland.; ^3^ Swiss Health Promotion Foundation, Bern, Switzerland.

**Keywords:** Health Policy, Health Promotion, Disease Prevention, Health Priorities

## Abstract

In Switzerland, the federal authorities, the cantons, and the communes share the responsibility of healthcare, disease prevention and health promotion policies. Yet, the cantons are in most health matters independent in their decisions, thus defining as a matter of fact their own health priorities.

We examined and analysed the content of the disease prevention and health promotion plans elaborated during the last decade in six French-speaking cantons with different political contexts and resources, but quite similar population health data, in order to identify the set health priorities.

The plans appear significantly inhomogeneous in their structure, scope and priorities. Most of the formal documents are short, in the 16 to 40 pages range. Core values such as equity, solidarity and sustainability are explicitly put forward in 2/6 cantonal plans. Priority health issues shared by all 6 cantons are "physical activity/sedentariness" and "nutrition/food." Mental health is explicitly mentioned in 5 cantonal plans, whereas tobacco and alcohol consumptions are mentioned 4 times.

Less attention has been given to topics that appear as major public health challenges at present and in the future in Switzerland, eg, ageing of the population, rise of social inequalities, increase of vulnerable populations. Little attention has also been paid to issues like domestic violence or healthy work environments. Despite some heterogeneity, there is a common base that should make inter-cantonal collaborations possible and coordination with national strategies easily feasible.

## Introduction

Defining and implementing health priorities is, at least in a democratic society, a complex process and a difficult one.^1^ The numerous stakeholders of the health sector may indeed have diverging interests and therefore support different options or approaches.^2^ Some argue that efficiency comes first because of the limited resources, while others push for more equity and solidarity^3^; some support technical solutions such as the Program Budgeting and Marginal Analysis (PBMA),^4^ while others plead for entrusting a group of experts, who take into account the existing empirical evidence^5^ when setting priorities; finally, some plead for engaging target populations in the process, in order to make public health services more relevant for them.^6^

In Switzerland, the federal authorities, the cantons, and the communes share the responsibility of healthcare, disease prevention and health promotion policies, yet the cantons are in most health matters independent in their decisions, thus defining as a matter of fact their own health policies.^7^ Over the years, significant attention was paid to improve and strengthen the collaboration between federal and cantonal health authorities as well as among cantons. In the process of a new federal law regulating mandatory insurance coverage for disease,^8^ an important step was taken when the Swiss Health Promotion Foundation was created to coordinate health promotion activities at the national level.^9^ Yet, a setback took place in regard to the central coordination when the parliament did not vote in favour of a national law on prevention that aimed to establish a National Health Institute, which was supposed to become the leading institution in disease prevention and health promotion as well as in public health research.^10^ The refusal was partially explained as a rejection to accept any restriction of cantonal independence. Nevertheless, with growing challenges the Swiss health system is facing (aging of the population, non-communicable chronic disease on the rise, costs increase), federal authorities have started to define sectorial health strategies,^11-13^ the Swiss Health Promotion Foundation being an associated partner regarding the NCDs strategy.^12^ The cantons, facing the same challenges, have besides planning healthcare, elaborated disease prevention and health promotion plans in order to tackle priority health issues.

We wondered which were the health priorities set in those plans, how they had been defined, whether those priorities reflected the health problems responsible for most of the burden of disease encountered in Switzerland and whether those plans share common aspects in terms of values, priorities and programs, which could eventually facilitate inter-cantonal collaborations and possibly would make adhesion to national strategies easier. We address these questions in the present paper.

## Methods

We examined and assessed disease prevention and health promotion strategic plans of the six French speaking Swiss cantons for declarative aspects for identified public health priorities, values underlying these priorities, and for selected disease prevention and health promotion intervention programs during the academic year 2013-2014^ [1] ^. The cantons considered were Fribourg, Geneva, Jura, Neuchâtel, Valais, and Vaud. The documents studied span the period 2003–2011. Discourse analysis was used to identify the values behind the health policies.^14^ We chose to use the values defined by the Commission of the European Communities in its White paper, ‘Together for health: a strategic approach for the EU 2008-2013’^15^ as the core public health values, ie, universality, access to good quality care/prevention, equity, solidarity, gender equality, citizens’ empowerment/health literacy, reducing inequalities in health, scientific evidence. We focused on public health problems identified as priorities by the various French-speaking cantons and, consequently, on intervention programs proposed by their respective strategic plans?

## Results

### Background Population Data

The French-speaking cantons considered in this study (Fribourg, Geneva, Jura, Neuchâtel, Valais, Vaud) share quite similar population health data.^16^ However they differ widely in terms of size, urban/rural share, economic specialization, level and sources of income: eg, population size varying from roughly 70 000 (canton Jura) to roughly 700 000 (canton Vaud); eg, net per capita annual income of 38 000 CHF (canton Valais) to 62 000 CHF (canton Geneva).^17^

### Planning Cycles

The documents studied span the period 2003–2011. The planning cycles covered vary significantly: one canton proposes a “multiannual” strategy, another does not specify the validity period. Four others propose plans for periods ranging from three to five years. In most cases the timing corresponds to local legislature cycles. A constant element is the wide variety of partners involved in the implementation of most of the programs under consideration.

### Methods Employed to Set Priorities

The methods and resources engaged in the document elaboration and the priority determination vary widely: from a selection among a small group of top priorities previously determined by experts to the work of ad hoc commissions and expert groups of varying sizes. The recourse to the opinion of experts is predominant, while the recourse to open, public, participative consultation processes still appears quite limited.

### Length and Scope of the Documents

Most of the formal documents are short, in the 16 to 40 pages range. None of the documents seeks to exhaustively encompass the whole domain of existing disease prevention and health promotion activities, and all recognize this fact.

The scope of the plans may be defined by ad hoc selection, by the legal perimeter of action for the offices involved, and/or by what appears relevant for priority actions. Priority status is repeatedly stated as non-competitive with existing programs. The plans primarily act as a framework for existing programs and for programs to be developed, thus also providing additional funding.

### Core Values and Data Sources

Two out of six cantons chose to explicitly, though briefly, state the core values of their health prevention and promotion plans: “*solidarity, equity, individual and collective responsibility*” in one case, “*sustainability, equality of chances, empowerment, cooperation, evaluation*” in the other. Indirect sources quoted are: the Ottawa Charter^18^ (2 cantons), the World Health Organization (WHO) Health for All objectives^19^ (3 cantons).

The most frequently referenced data sources are the OBSAN surveys for the general population^16^ and well-established surveys for the health of children and adolescents (HSBC, SMASH).^20^ Other sources quoted include the Swiss Health Promotion Foundation, local laws, and a federally financed study on the economic impact of disease prevention and health promotion measures.^21^

### The Continuity of Existing Programs

All the documents we examined underline the need for stable programs running on the long term, eg, organized breast cancer screening programme. Those are seldom examined in detail. None attempts to draw an inventory of existing programs, nor to give an exhaustive picture of what is being done in the canton: “exhaustiveness in this area is impossible to achieve,” acknowledges a cantonal master plan.

### Frequency of Priority Themes

In Table are presented the main health promotion and disease prevention priority themes in the six cantons.

Promoting healthy eating and physical activity are addressed together and identified as priorities in all cantonal plans. The “Healthy Body Weight Program” of the Swiss Health Promotion Foundation is widely acknowledged.^22^ The “Green Fork” labelling program is also mentioned in all cases.^23^ Regarding physical activities two main trends emerge, some cantons emphasizing the promotion and support of popular sport and sports clubs, while others emphasize the promotion of physical activity and soft mobility through structural means, such as the development of bike paths and infrastructure. The specific needs of elderly people in terms of physical activity and nutrition are explicitly mentioned only in a minority of cases.

Mental health appears to be a priority in 5 out of 6 cases. Prevention of suicide and improved detection of depression receive particular attention with proposed programs such as “Alliance Against Depression,”^24^ but some cantons are also developing broader initiatives addressing “existential distress.” In the most recent cantonal plans, there are references to the initiative of the Swiss Health Promotion Foundation “Mental Health and Stress.”^25^

The prevention of addictions (Alcohol, tobacco and other addictive substances) also appears to be at the centre of interest, but the emphases and sensitivities vary considerably according to the cantons. The national health policy documents are mentioned with some frequency, but several cantons feel the need to differentiate between approaches.^26,27^ The range of measures proposed for alcohol is very broad, from sensitization/general information or to targeted measures for the protection of minors, to the detection of excessive or risky consumption. However, effective measures such as test shopping have not been adopted everywhere and neither are the concepts of youth protection.

While the six cantons studied have at least one well-established screening program for cancer screening (breast cancer), four planning documents mention this. The fight against cancer appears to be a priority objective to be developed and/or strengthened in the two most recent cantonal plans, which also mention primary prevention by vaccination against human papillomavirus (HPV). One of these plans explicitly considers the development of colon cancer screening.

Infectious disease prevention is mentioned in three cantonal plans including vaccination campaigns (influenza for example), prevention of HIV and sexually transmitted disease (STD) and measures to fight multi-resistant organisms.

The prevention of accidents appears in two cantonal plans. The emphasis is specifically on road accident prevention activities. Only one cantonal plan mentions the campaigns of the National Accident Insurance SUVA focusing of domestic, leisure and sport accidents.^28^

Health of the Elderly is developed in 2 plans, referring to demographic ageing.^29^ Other topics (see Table) are only mentioned once.

**Table:** Main Priority Themes of Disease Prevention and Health Promotion Plans in Six Swiss Cantons

**Themes**	**Priorities for Action Mentioned by the Cantonal Plans**
Physical activity	6/6
Nutrition and food	6/6
Mental health	5/6
Tobacco	4/6
Alcohol	4/6
Cannabis and other addictions	4/6
Breast cancer	4/6
Environmental and social conditions	3/6
Infectious diseases and vaccinations	3/6
Accidents and violence	2/6
Health of the elderly	2/6
Health information services	1/6
Health at work	1/6
Health professionals capacity building	1/6
Information and communication	1/6
Sustainable development	1/6

## Discussion

We had four areas of focus when we examined the strategic health plans of French speaking Swiss cantons. We wanted to know which the identified health priorities were in the plans; how they had been defined; whether those priorities reflected the health problems responsible for most of the burden of disease encountered in Switzerland; and whether those plans share common aspects in terms of values, priorities, and programs. In a regional context these questions are of some importance and relevance since, depending on the observed similitudes and differences recommendations to more collaboration and coordination could be done.

The form and content of the various cantonal plans documents appear quite inhomogeneous, yet the chosen approaches in establishing those plans are quite similar, ie, expert committees elaborated the plans based on health data collected locally/regionally/nationally; some differences were noted, such as the size and the composition of the committees (limited to health professionals, including administrative/financial state staff). In the process of defining health priorities, none of the expert groups seems to have adopted a multi-criteria decision analysis approach, despite the recommendations by some authors due to the complexity of the issues and the multiplicity of the stakeholders.^1^ Yet the expert committee approach, which was adopted by the cantons in order to establish their strategic health plans, yielded similar results as Schopper et al, who established health priorities for the state of Geneva through a series of specific surveys and studies (potential years of life lost, disability adjusted years of life lost, Delphi survey among health professionals and the general public) some fifteen years ago.^30^

When stated (5 out of 6), the plans share common values, such as solidarity, equity, individual and collective responsibility, equality of chances, empowerment, cooperation, evaluation, values put forward in the Ottawa Charter,^18^ the Health for All framework of WHO^19^ and the EU Together for Health Strategy White Paper^15^ and supported in the scientific literature.^31^

The examined cantonal health plans have essentially identified the major health problems Swiss health authorities are facing,^1,2,6^ and proposed intervention in order to reduce those problems. Indeed, the cantonal plans identify and target major risk factors such as sedentariness, eating habits, smoking and alcohol abuse, as well as major health problems, such as mental health/mental disorders and cancers.^32^ When comparing the public health priorities set by each canton to the health data available at the national level in Switzerland,^16,32,33^ we observe that the emphasis on physical activity and healthy nutrition programs are likely a result of high incidence rates in cardiovascular disease in the respective cantons. Cardiovascular diseases are the primary cause of death in Switzerland and the top ranking cause of years of life lost (YLLs) and of disability adjusted life years (DALYs).^34^ The emphasis on physical activity could also possibly be related to chronic back pain, one of the top 5 causes of years lived with disabilities (YLDs).^34^ The emphasis on healthy nutrition is likely based on statistics implying that 11.5% of DALYs are attributable to dietary risk behaviours and 7.5% to high body mass index in Switzerland.^34^

The interest in mental health promotion (5 of 6 cantons) likely aims to address the heavy burden of depression – the leading cause of YLDs in Switzerland – and suicide, as 10% of Swiss citizenscommit one or more suicide attempts during their lifetime.^34-36^ Four out of six cantons mention cancer prevention programs as of high priority: indeed, all the French-speaking cantons have implemented organized breast cancer screening programs with some success over the past 20 years.^37^ However, at present, there is some debate about their relevance and the Swiss Medical Board has proposed to abolish those programs, questioning its cost-effectiveness.^38^

Interestingly, the fight against smoking is only identified as priority in 4 cantons. Smoke-free programs might deserve more attention, since smoking is credited responsible for 10% of the burden of disease and 25% of the population over the age of 15 years still smokes.^39,40^ This is also the case of the fight against alcohol abuse mentioned in 4 cantons: 5.5% of the population of the 6 cantons is at medium to high risk (daily consumption ≥20 g alcohol) with peaks among young male adults (8%).^39^ Percentages of harmful alcohol consumption jump to 30% among young male adults when the maximum alcohol consumption on a single occasion (>9 drinks) over the last 12 months is considered.^39^

The most cited fields of interventions in the cantonal health plans come close to evidence-based interventions with the best cost-effectiveness ratio for increasing health status of a population recommended by the WHO (reducing tobacco use, promoting physical activity, reducing harmful alcohol use, promoting healthy diets),^40^ if and only if the proposed programs will effectively be implemented.

Less attention has been given to topics that appear as major public health challenges at present and in the future in Switzerland, eg, ageing of the population, rise of social inequalities, increase of vulnerable populations; little attention also to issues like domestic violence, or healthy work environments despite their public health relevance. Indeed, the Swiss population is in a rapid ageing process, with the proportion of people aged ≥65 in comparison to people aged 20-64 expected to increase from 27.5% in 2010 to 43% by 2030; furthermore the proportion of the very old (≥80) as compared to the people age 65-79 is expected to increase by 75%.^41^ Vulnerable populations in the society need more attention, their health being especially at risk: indeed, studies have shown a higher prevalence of various disease, such as depression, musculoskeletal problems, renal disease and chronic bronchitis among immigrant populations, compared to the native population, and an increasing gap between the two populations with age.^42^ As for domestic violence, it remains a subject little taken into consideration by those responsible for the health of the population, even though its prevalence is high and the risk to the health of the victims and their entourage is not negligible (roughly 20% of women are victims of domestic violence and 1 women dies every other week).^43^ Eventually, it should be noted that according to a study “health at work is the great forgotten one” by representatives of health administrations and institutions in the cantons.^44^

## Conclusion

The analysis of the contents of the cantonal disease prevention and health promotion plans, and the adopted approach through expert committees for defining those plans, allows us to conclude that there is some regional coherence between the cantons and with national programs and international recommendations. Despite some heterogeneity, there is a common base that should make inter-cantonal collaborations possible and coordination with national strategies easily feasible. However, some public health issues may not have received the attention they deserved given their importance in terms of population health and social cohesion. But setting priorities for disease prevention and health promotion remains a delicate exercise given the number of stakeholders likely to be affected.

## Ethical issues

Not applicable.

## Competing interests

Authors declare that they have no competing interests.

## Authors’ contributions

Data analysis (CP, JS, NC, ZR, TM); Draft writing (CP, JS, TM); Text revision (JS, CP).

## Authors’ affiliations

^1^University of Fribourg, Fribourg, Switzerland. ^2^Institute of Global Health, University of Geneva, Geneva, Switzerland. ^3^Swiss Health Promotion Foundation, Bern, Switzerland.

## Endnotes


[1] Cantonal plans and drafts examined:

Programme cadre en promotion de la santé et prévention pour le Canton du Valais, 2011-2014. Service de la santé publique (VS), 2011.

Plan cantonal de promotion de la santé et de prévention 2007-2011. Service de la santé publique (FR), 2006.

Programme pluriannuel de prévention et promotion de la santé. République et Canton du Jura, Service de la santé publique (JR), 2003.

Prévention et promotion de la santé. Plan directeur. République et Canton de Neuchâtel. Service de la santé publique (NE), 2009.

Rapport du Conseil d’Etat sur la politique sanitaire 2008-2012. Canton de Vaud (VD), 2007.

Plan cantonal genevois de promotion de santé et de prévention 2007/2010. Direction générale de la santé, 2007.

